# RASSF6 downregulation promotes the epithelial-mesenchymal transition and predicts poor prognosis in colorectal cancer

**DOI:** 10.18632/oncotarget.19181

**Published:** 2017-07-12

**Authors:** Rui Zhou, Lin Qiu, Xiaolong Liu, Lijuan Ling, Ninglei Li, Kun Zhou, Jingbo Sun, Jian Yan, Canliang Tan, Xiaoping Huang, Luzhe Han, Liangchun Yin, Gang Xiao, Lixin Liu

**Affiliations:** ^1^ Department of General Surgery, The Third Affiliated Hospital of Southern Medical University, Guangzhou, China; ^2^ Department of Hematology/Oncology, Guangzhou Women and Children's Medical Center, Guangzhou Medical University, Guangzhou, China

**Keywords:** RASSF6, Wnt, EMT, colorectal cancer, metastasis

## Abstract

Distant metastasis is the primary barrier for the successful treatment of patients with colorectal cancer, and thus, searching for new therapeutic targets by further exploring the molecular mechanisms of colorectal cancer metastasis is important. In this study, we investigated the biological and clinical significance of RASSF6 in colorectal cancer as well as the underlying molecular mechanisms. We found that low RASSF6 expression corresponds to a poor prognosis in colorectal cancer patients, and low RASSF6 expression is distinctly associated with tumour progression. Our *in vitro* analysis revealed that RASSF6 suppresses the proliferation and metastasis of DLD1 cells, and RASSF6 knockdown in HCT116 cells confirmed these observations. Our mechanistic investigation revealed that RASSF6 inhibits the expression of the classical target genes of Wnt signalling, as demonstrated by the reduced expression of TCF1, c-Jun, and c-Myc in RASSF6-overexpressing DLD1 stable cell lines. Furthermore, we show that RASSF6 functions as a negative regulator of the epithelial-mesenchymal transition; the expression levels of the epithelial markers ZO-1 and E-cadherin were increased, while the expression level of the mesenchymal marker Snail was decreased in a RASSF6-overexpressing DLD1 cell line. Additionally, rescue assays revealed that the activation of Wnt signalling by LiCl treatment impaired the inhibitory effect of RASSF6 on the proliferation and metastasis of colorectal cancer cells, which implies that RASSF6 suppresses the tumorigenicity of colorectal cancer cells at least in part through inhibiting Wnt signalling pathway. Collectively, these findings provide new perspectives for the future study of RASSF6 as a therapeutic target for colorectal cancer.

## INTRODUCTION

Approximately 700,000 patients die from colorectal cancer (CRC) annually, which corresponds to the fourth most deadly cancer worldwide [1]. Despite an increased awareness of the pathogenic risk of CRC and advances in therapeutic strategies, CRC remains a major cause of cancer morbidity and remains the second leading cause of cancer death in China [2, 3]. Among CRC patients with distant metastasis, the five-year survival rate is lower than 10% [4], and metastasis causes as much as 90% of cancer-associated mortalities [5]. Distant metastasis remains a key barrier to therapy in CRC patients, which makes it imperative to search for new therapeutic targets by further exploring the underlying mechanisms of CRC metastasis.

The epithelial-mesenchymal transition (EMT) is a complicated process that endows epithelial cells with mesenchymal properties by altering their morphology, adhesion, and migratory capacity [6]. When EMT occurs in benign tumour cells, these cells become more effective at invading surrounding tissues and metastasizing to distant sites [7, 8]. Compelling evidence has demonstrated that the efficient suppression of EMT can inhibit CRC metastasis and improve the survival rates of CRC patients [9]. Ras association domain family (RASSF) 6 is a member of the Ras association domain family, which consists of six classical RASSFs (RASSF1 to RASSF6) and the N-terminal RASSFs (RASSF7 to RASSF10) with a N-terminal Ras association domain [10]. Classical RASSFs (RASSF1 to RASSF6) encode a C-terminal RA and SARAH domain, which encode tumor suppressors, involved in cell cycle regulation, microtubule stability and apoptosis. However, unlike other members of RASSFs, RASSF6 does not bind Ki-Ras or any of the other Ras proteins [11–14]. RASSF6 has been studied in multiple several tumors, including gastric cancer, melanoma, nasopharyngeal carcinoma, and pancreatic ductal adenocarcinoma [15–18]. RASSF6 inhibits invasion in melanoma cells [17] and enhances the sensitivity of highly metastatic nasopharyngeal carcinoma cells to cisplatin. Accumulating evidence that low RASSF6 expression predicates poor prognosis in multiple gastrointestinal tumors, such as gastric cancer, hepatocellular carcinoma, pancreatic ductal adenocarcinoma. However, there is no evidence with respect to the prognostic value of RASSF6 expression in patients with CRC [15, 16]. Despite a previous study indicating that RASSF6 inhibits cell growth and migration in HT29 and Lovo cell lines [19], further underlying mechanisms is needed to determine how RASSF6 suppress proliferation and metastasis in CRC, and to determine whether the expression level of RASSF6 correlates with the prognosis of CRC patients.

The Wnt signalling pathway is dysregulated in more than 90% of colorectal cancers, which contributes to the progression of colorectal cancer [20, 21]. Previous studies have demonstrated that the dysregulation of the Wnt pathway is involved in many types malignant tumours and is an important regulator of biological processes, such as cell growth, migration and EMT process [22, 23]. Genetic or epigenetic alterations in the Wnt signalling components results in aberrant activation of target genes and promotes cellular proliferation and invasion [24]. Given the well-established role of Wnt signalling in the regulation of CRC tumour progression, we hypothesized that RASSF6 influences CRC cell function through the Wnt signalling pathway.

In the present study, we first determined the expression level of RASSF6 in eight CRC cell lines and in patient tissues, and we then investigated potential mechanisms to elucidate how RASSF6 inhibits CRC progression. Here, we report for the first time that RASSF6 inhibits the EMT process and CRC cell function and tumour progression by suppressing the Wnt signalling pathway.

## RESULTS

### RASSF6 is downregulated in CRC cell lines and patient tumour tissues

**Figure 1:**
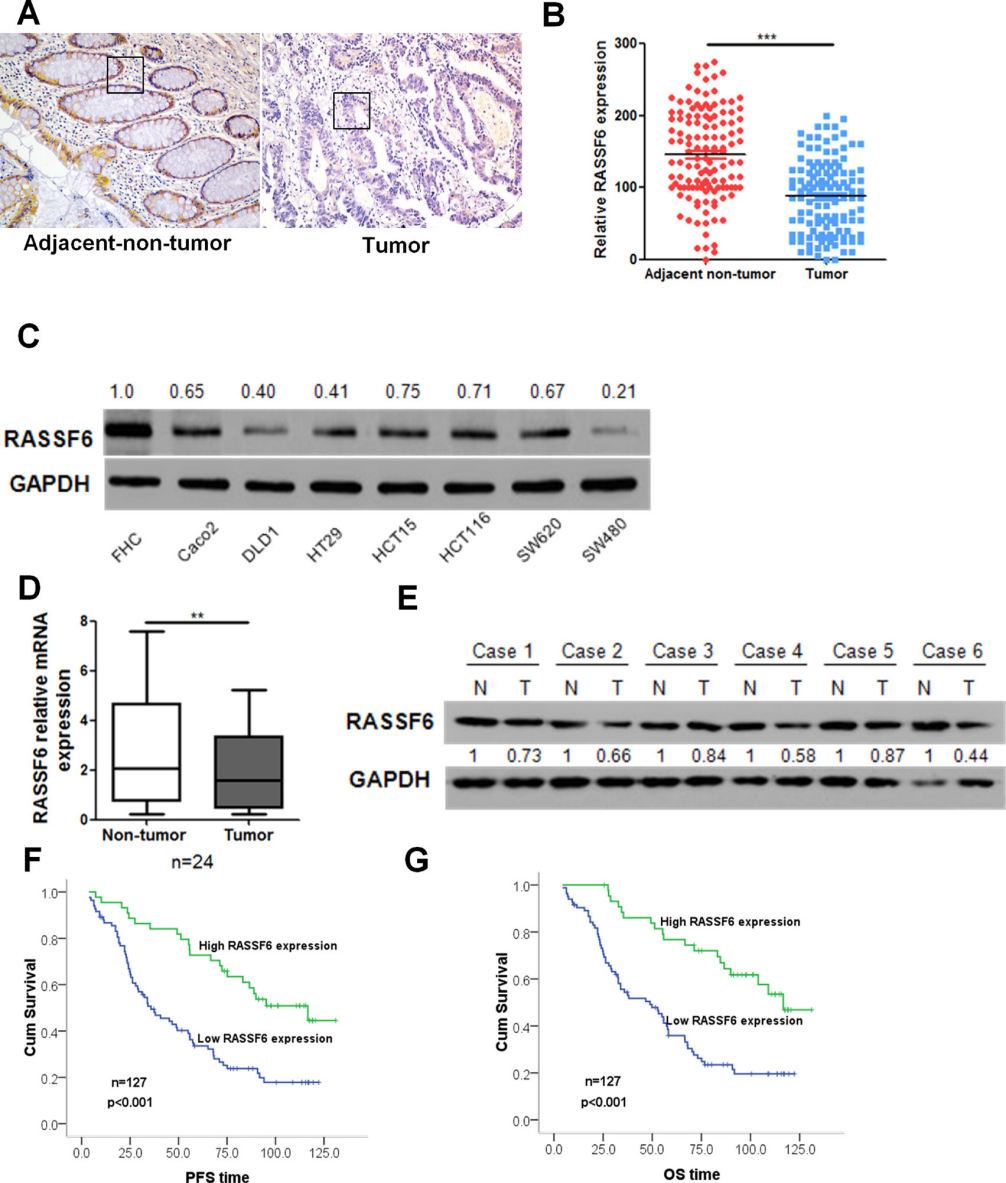
RASSF6 is downregulated in CRC cell lines and patient tumour tissues and has prognostic value in patients with CRC (**A**–**B**) RASSF6 expression was detected by IHC analysis in CRC tissues and adjacent normal mucosal tissues; each point in the graph represents the RASSF6 expression H-score of an individual patient tumour (*n* = 127, ***p < 0.001, paired Student's *t*-test). (**C**) The expression of RASSF6 in the normal colon epithelial cell line FHC and seven colorectal cancer cell lines was examined by western blotting. GAPDH was used as a reference control. (**D**) The relative expression level of RASSF6 was examined by q-PCR in 24 CRC tissues (Tumor) and their paired normal mucosal tissues (Non-tumor). GAPDH was used as an endogenous control. **p < 0.01. (**E**) RASSF6 expression at the protein level was detected by western blot analysis in 6 pairs of CRC tissues and paired normal mucosal tissues. GAPDH was used as a loading control. (**F**, **G**) Kaplan-Meier survival analysis of the association between RASSF6 expression and PFS or OS of CRC patients (log-rank test).

To investigate the expression levels of RASSF6 in CRC, we first examined the expression of RASSF6 in CRC patient tissues. IHC was performed using paraffin-embedded tissue sections (*n* = 127) of histopathologically confirmed CRC. Our results indicated that the expression of RASSF6 is significantly decreased in tumour tissues compared with its expression in paired adjacent non-tumour tissues (*p* < 0.001, Figure 1A, 1B). To further explore the RASSF6 expression pattern in CRC, we determined the expression of RASSF6 by western blotting in eight CRC cell lines. As shown in Figure 1C, our results indicate that RASSF6 expression is downregulated in CRC cell lines compared with its expression in normal epithelial cell line FHC. Moreover, the expression of RASSF6 also significantly decreased in tumour tissues compared with its expression in paired adjacent non-tumour tissues at both mRNA and protein level (Figure 1D, 1E). These results demonstrate that RASSF6 expression is downregulated in CRC, which implies that RASSF6 may act as a tumour suppressor in CRC.

### RASSF6 expression is associated with clinicopathological characteristics

To further investigate the significance of RASSF6 expression in CRC, the correlations between RASSF6 expression and clinicopathological features of CRC patients were analysed. The clinical characteristics of the patients are summarized in Table 1. The levels of RASSF6 immunoreactivity varied between tumour tissue samples and the adjacent non-tumour tissue samples. As shown in Table 1, low RASSF6 expression was detected in 83 (65.3%) of the resected tumour tissue samples, whereas the remaining 44 cases (34.6%) displayed high levels of RASSF6 expression. Furthermore, low RASSF6 expression was significantly associated with tumour size (*p* = 0.042), TNM stage (*p* < 0.001), and the presence of distant metastases (*p* < 0.001). A low level of RASFF6 expression indicated a larger tumor size, more advanced TNM stage, and the presence of lymph node and distant metastasis. No other significant correlations were observed between RASSF6 expression level and age, gender, or preoperative carcinoembryonic antigen (CEA) expression level. Collectively, our results provide powerful evidence that low RASSF6 expression is associated with CRC proliferation, metastasis, and progression.

**Table 1:** Clinicopathological findings and correlation with RASSF6 expression

Variables	*N* (%)	RASSF6-low (%)	RASSF6-high (%)	*P* value
Total cases	127	83 (65.3)	44 (34.6)	
Age (years)				
< 60	61 (48.0)	35 (27.6)	26 (20.5)	0.10
≥ 60	66 (52.0)	48 (37.8)	18 (14.2)	
Gender				
Male	69 (54.3)	46 (36.2)	23 (18.1)	0.852
Female	58 (45.7)	37 (29.1)	21 (16.5)	
Tumour Size (cm)				
< 5	59 (46.5)	33 (26.0)	26 (20.5)	0.042^b^
≥ 5	68 (53.5)	50 (39.4)	18 (14.2)	
AJCC/TNM stage^a^				
I–II	51 (40.2)	20 (15.7)	31 (24.4)	< 0.001^b^
III–IV	76 (59.8)	63 (49.6)	13 (10.2)	
Lymph node status				
< 1	51 (40.2)	20 (15.7)	31 (24.4)	< 0.001^b^
≥ 1	76 (59.8)	63 (49.6)	13 (10.2)	
Distant metastasis				
No metastasis	74 (58.3)	38 (29.9)	36 (28.3)	< 0.001^b^
Metastasis	53 (41.7)	45 (35.4)	8 (6.3)	
Preoperative CEA (ng/ml)				
< 5	65 (51.1)	40 (31.5)	25 (19.7)	0.456
≥ 5	62 (48.8)	43 (33.8)	19 (15.0)	

NOTE: The numbers in parentheses indicate the percentages of tumours with a specific clinical or pathological feature for a given RASSF6 subtype.

Abbreviations: CEA, carcino-embryonic antigen.

^a^According to the 7th Edition of the AJCC Cancer Staging Manual.

^b^Statistically significant.

### Low RASSF6 expression predicts poor prognosis in CRC patients

To gain further insight into the prognostic value of RASSF6 expression in patients with CRC, the relationship between RASSF6 expression and patient progression-free survival (PFS) and overall survival (OS) were analysed. As shown in Figure 1F and 1G, the mean value of PFS (52.18 vs. 93.06) and OS (55.70 vs. 99.32) were significantly lower in the RASSF6 low-expression group than that in the RASSF6 high-expression group, and RASSF6 expression was significantly associated with PFS (*p* < 0.001) and OS (*p* < 0.001). Furthermore, Cox proportional hazard regression analysis indicated that RASSF6 expression behaves as an independent prognostic factor for PFS (*p* = 0.026, HR = 0.52, 95% CI 0.30–0.93) and OS (*p* = 0.03, HR = 0.52, 95% CI 0.29–0.94; Table 2) in CRC.

**Table 2:** Multivariate analysis for PFS and OS

Variable	PFS HR (95% CI)	*P* value	OS HR (95% CI)	*P* value
Age (year, < 60 vs ≥ 60)	0.70 (0.44–1.12)	0.14	0.71 (0.44–1.17)	0.18
Gender (male vs female)	0.89 (0.57–1.41)	0.63	0.92 (0.57–1.48)	0.73
CEA (ng/ml, < 5 vs ≥ 5)	1.28 (0.82–2.01)	0.28	1.41 (0.89–2.23)	0.15
Tumour size (cm, < 5 vs ≥ 5)	0.78 (0.49–1.26)	0.31	0.91 (0.56–1.48)	0.71
TNM stage (I–II vs III–IV)	3.65 (1.08–12.29)	0.037^a^	3.62 (1.02–12.81)	0.046^a^
Lymph node status (< 1 vs ≥ 1)	4.07 (1.11–14.96)	0.034^a^	6.88 (1.6–29.48)	0.009^a^
Distant metastasis (no vs yes)	2.21 (1.25–3.90)	0.007^a^	2.02 (1.14–3.56)	0.015^a^
RASSF6 (low vs high)	0.52 (0.30–0.93)	0.026^a^	0.52 (0.29–0.94)	0.030^a^

PFS progression-free survival, OS overall survival, HR hazard ratio, CI confidence interval.

^a^Statistically significant.

### RASSF6 inhibits metastasis, invasion, and proliferation in CRC cell lines

**Figure 2:**
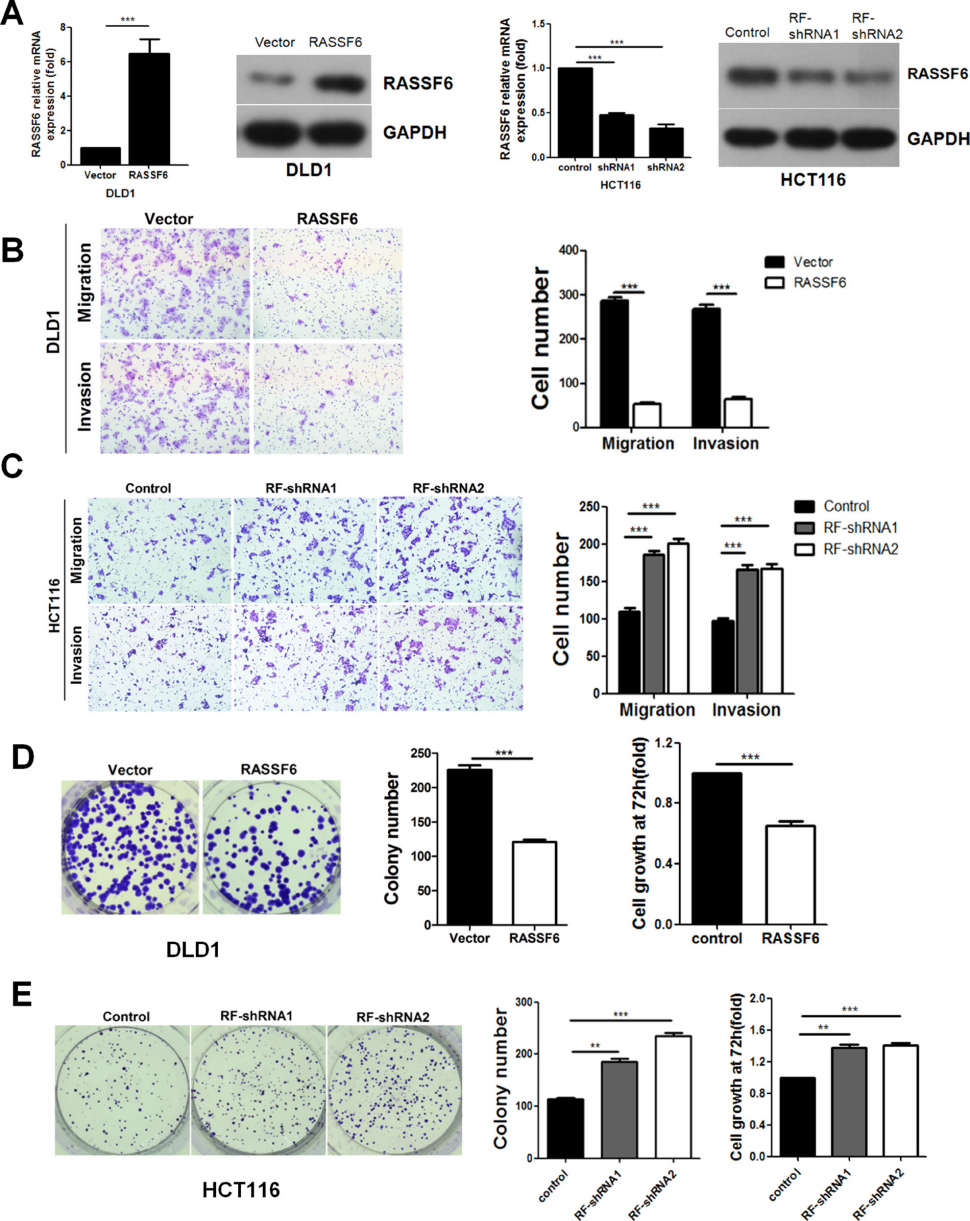
The effects of RASSF6 on cell growth and motility in colorectal cancer cell lines (**A**) The efficient overexpression or suppression of RASSF6 was confirmed by q-PCR and western blotting. (**B**–**C**) Representative images show the migration and invasion abilities of DLD1 and HCT116 cells with RASSF6 stably overexpressed or knocked down. The number of cells was quantified. ***p < 0.001, Student's *t*-test. (**D**–**E**) Colony formation assays were performed, and MTT assays were conducted 72 h after the cells were plated. Representative images show the proliferation ability of DLD1 and HCT116 cells with RASSF6 stably overexpressed or knocked down. The number of colonies was quantified. These above experiments were repeated at least three times. Error bars, mean ± SD, **p < 0.01 ***p < 0.001 using Student's *t*-test.

Given that RASSF6 is negatively associated with distant metastasis in CRC clinical samples, we next sought to investigate the effects of RASSF6 on metastasis and invasion in CRC cell lines. To address this issue, we established the HCT116 stable cell lines RF-shRNA1 and RF-shRNA2 to suppress RASSF6 expression along with a control stable cell line shNC. Additionally, a DLD1 cell line stably overexpressing RASSF6 and a control DLD1 cell line, named RASSF6 and Vector, respectively, were generated. Both mRNA and protein expression levels of RASSF6 were confirmed by q-PCR and western blotting (Figure 2A), after which transwell migration and Matrigel invasion assays were conducted. The results of our analyses showed that the overexpression of RASSF6 distinctly suppresses the migration and invasion capacity of DLD1 cells (*p* < 0.001, Figure 2B), and correspondingly, the HCT116 cells with stable RASSF6 knockdown (RF-shRNA1 and RF-shRNA2) exhibited enhanced capacity for metastasis and invasion (*p* < 0.001, Figure 2C). Moreover, results above were also confirmed in HCT15 cell lines (Supplementary Figure 1). These results suggest that RASSF6 is a metastasis suppressor in CRC cell lines.

We next examined the effect of RASSF6 on the proliferation of CRC cells using both MTT and colony formation assays. As shown in Figure 2D, RASSF6 upregulation in the RASSF6 DLD1 cells dramatically inhibits cell growth compared with the control group (*p* < 0.001), while the persistent suppression of RASSF6 significantly induces the proliferation capacity of CRC cells (*p* < 0.001, Figure 2E).

### RASSF6 suppresses the Wnt signalling pathway

**Figure 3:**
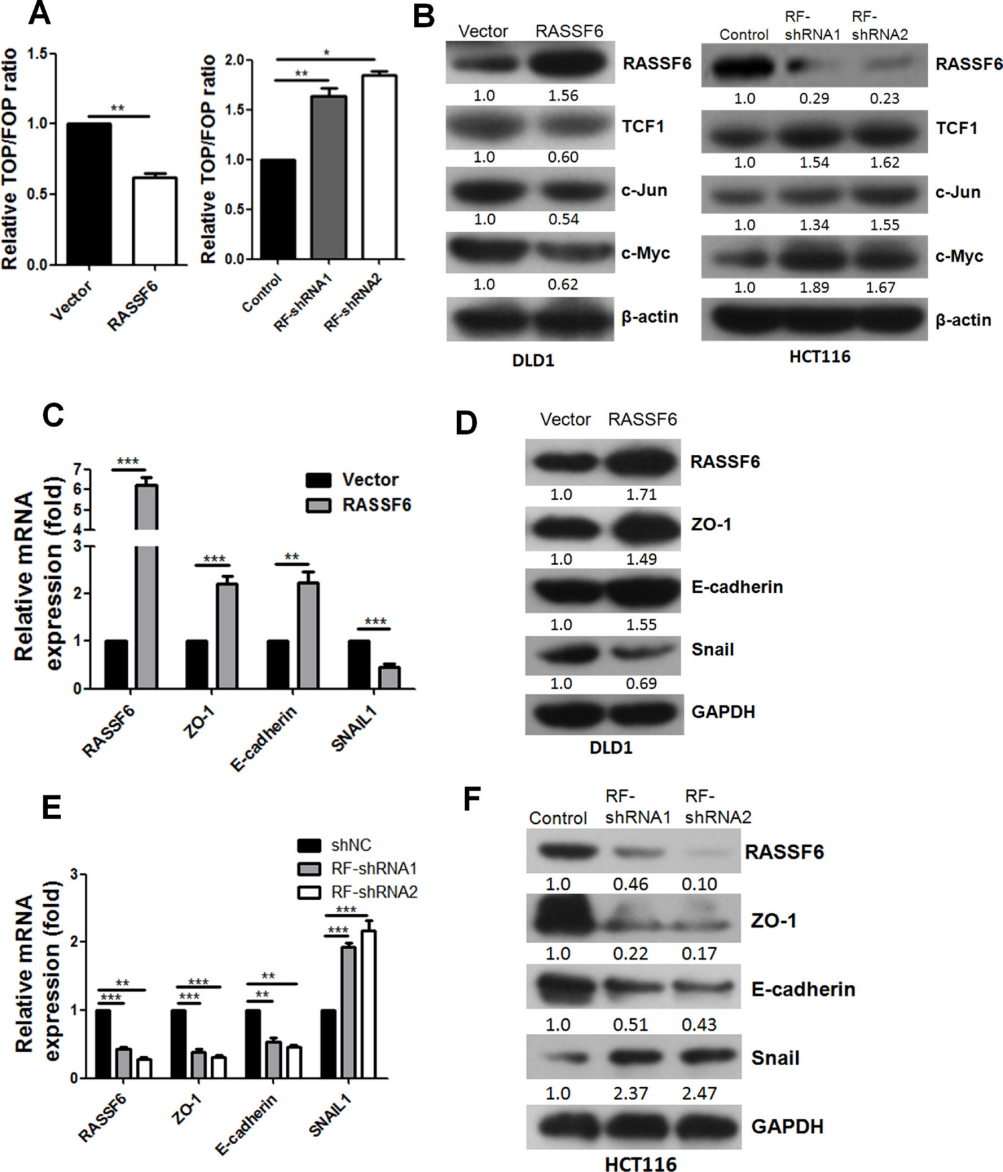
RASSF6 suppresses the Wnt signalling pathway and EMT (**A**) Effects of RASSF6 on TCF transcriptional activity in CRC cell lines. Means±SD of triplicate samples are shown. *p < 0.05, **p < 0.01. (**B**) Western blotting analysis of the expression of classical Wnt signalling target genes (TCF1, c-Jun, and c-Myc) in the indicated cells with RASSF6 stably overexpressed or knocked down. β-actin was used as an internal control. (**C**–**D**) Q-PCR and western blotting were conducted to detect the expression of EMT markers (ZO-1, E-cadherin, and Snail) in DLD1 stable cell lines. (**E**–**F**) Q-PCR and western blotting were performed to detect the expression of EMT (ZO-1, E-cadherin, and Snail) markers in HCT116 stable cell lines. Means ± SD of triplicate samples are shown. **p < 0.01, ***p < 0.001, Student's *t*-test.

Based on the above findings, we further investigated the potential mechanisms mediating RASSF6-regulated proliferation and metastasis in CRC. Due to the critical role of Wnt signalling in CRC, we first determined whether RASSF6 influences CRC function through Wnt signalling by conducting a TOP/FOP-Flash reporter assay. As shown in Figure 3A, the upregulation of RASSF6 caused a significant decrease in TOP-Flash activity (*p* < 0.01), and the silencing of RASSF6 remarkably induced TOP-Flash activity in CRC cell lines (*p* < 0.01). Moreover, changes in the expression levels of several classical Wnt signalling target genes were determined by western blotting. Interestingly, our results revealed that TCF1, c-Jun, and c-Myc were significantly downregulated in the RASSF6-overexpressing cell line and were correspondingly upregulated in RASSF6 stable knockdown HCT116 cells (Figure 3B), all of which strongly suggest that RASSF6 inhibits the activation of Wnt signalling pathway.

### RASSF6 is a negative regulator of the EMT process

Given that RASSF6 suppresses the migration and invasion of CRC cell lines, we next examined whether RASSF6 influences the EMT process in CRC. Consistent with our hypothesis, the mRNA and protein expression levels of epithelial markers ZO-1 and E-Cadherin distinctly increased in the RASSF6-overexpressing stable DLD1 cells, whereas the expression of the mesenchymal marker Snail dramatically decreased (Figure 3C, 3D). These results were further confirmed in the RASSF6 stable knockdown HCT116 cells (Figure 3E, 3F). Collectively, our results indicate that RASSF6 contributes to the inhibition of EMT in CRC cells, which at least partially accounts for the observation that RASSF6 overexpression suppresses metastasis and invasion in CRC cells.

### RASSF6 inhibits tumorigenesis in colorectal cancer cells via the Wnt signalling pathway

**Figure 4:**
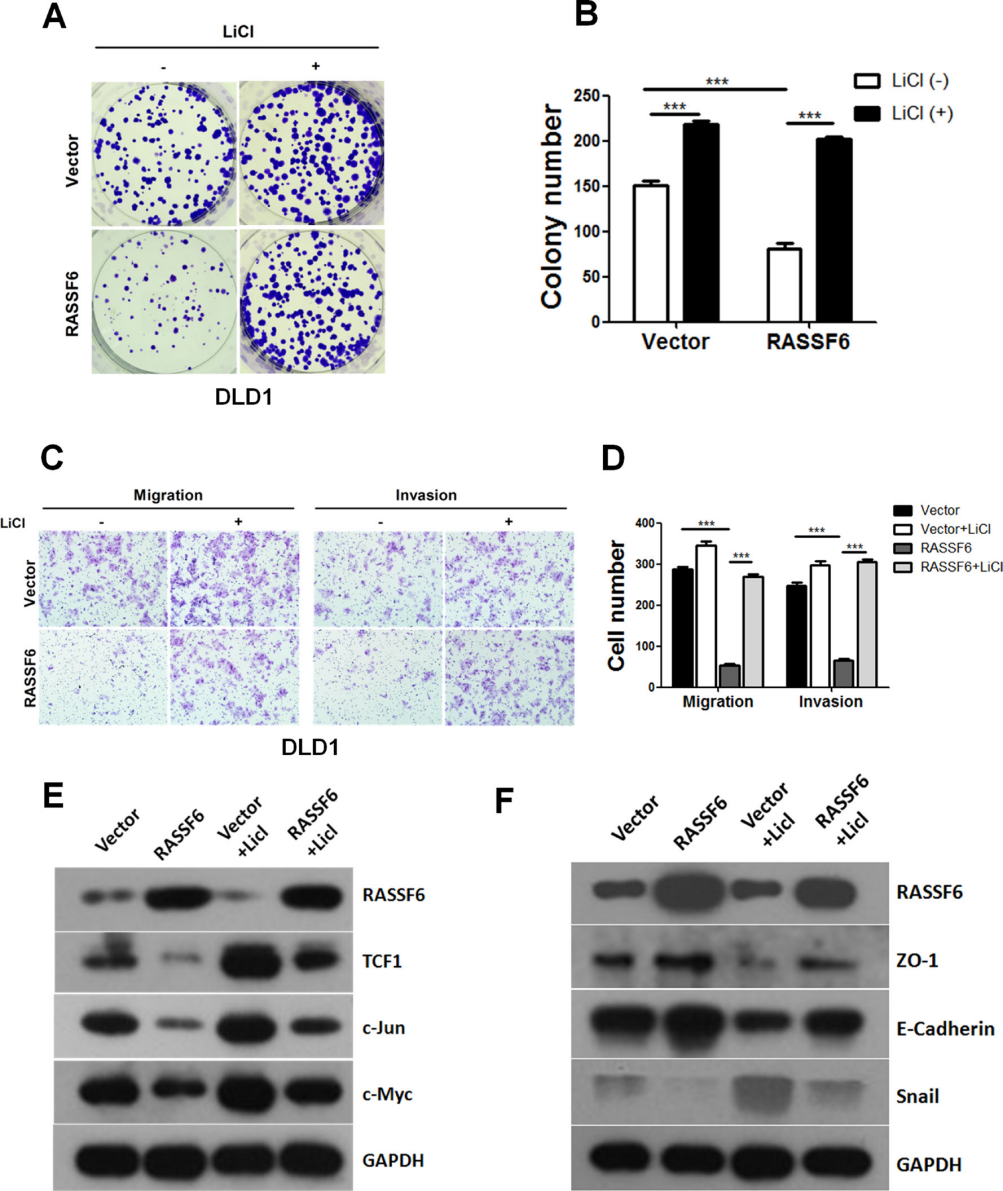
RASSF6 inhibits the tumorigenesis of colorectal cancer through the Wnt signalling pathway (**A**–**B**) The indicated cell lines were treated with LiCl. Representative images of colony formation rescue assays and quantification of the results are presented. Means ± SD of triplicate samples are shown. ***p < 0.001 based on Student's *t*-test. (**C**–**D**) The indicated cell lines were treated with LiCl, and migration and invasion transwell rescue assays were performed. Representative images of the results and quantification of the results are presented. Means ± SD of triplicate samples are shown. ***p < 0.001 based on Student's *t*-test. (**E**–**F**) The DLD1 stable cell lines (Vector, RASSF6) were treated with LiCl, and the cell lysates were collected for detection of the expression levels of Wnt targets or EMT markers by western blotting.

Based on our results showing that RASSF6 inhibits Wnt signalling, we wondered whether RASSF6 inhibits cell growth and motility in CRC cells by inactivating the Wnt signalling pathway. To validate our hypothesis, the stable DLD1 cell lines Vector and RASSF6 were treated with LiCl or PBS. Our results revealed that the inhibitory effects on proliferation caused by RASSF6 could be abolished upon LiCl treatment (Figure 4A, 4B). Additionally, transwell assays also indicated that LiCl reversed the inhibitory effect of RASSF6 overexpression on migration and invasion in DLD1 stable cells (Figure 4C, 4D). To further validate our observations, expression levels of the Wnt target genes and EMT markers noted above were examined by western blotting. As shown in Figure 4E and 4F, treatment with LiCl reversed the decreased expression of Wnt target genes caused by RASSF6 and specifically promoted EMT process, as the expression levels of E-cadherin and ZO-1 were significantly deceased, and the expression level of the mesenchymal marker Snail was increased. Taken together, our findings suggest that RASSF6 suppresses the tumorigenesis of CRC at least in part through the negative regulation of the Wnt signalling pathway.

### RASSF6 inhibits the growth and metastasis of CRC cells *in vivo*

**Figure 5:**
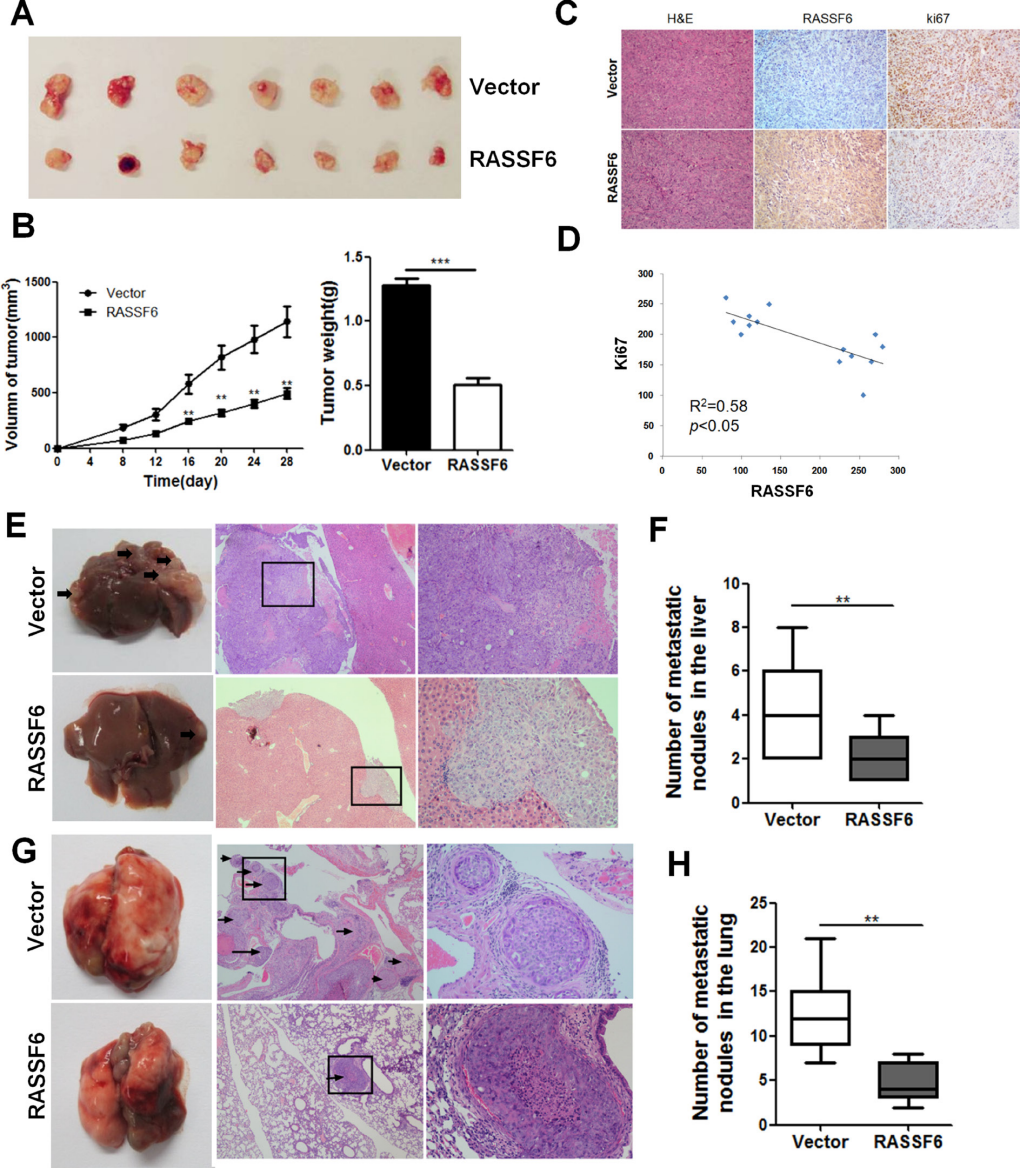
RASSF6 inhibits tumorigenesis in nude mice (**A**) Xenograft tumours were harvested 28 days after implantation. (**B**) Data points are presented as the means ± SD for tumour volumes, and the weights of the tumours were analysed. **p < 0.01. (**C**) The tumour sections were stained with H&E or were subjected to IHC staining using antibodies against RASSF6 and Ki-67 (200× magnification). (**D**) Correlations between the expression levels of RASSF6 and Ki67 were analysed in mouse tumour tissues using Pearson's test. (**E**, **F**) Hepatic metastasis model. The liver sections were subjected to H&E staining, and the metastatic nodules are indicated with arrows. The number of metastatic nodules in the liver specimens was analysed. **p < 0.01. (**G**, **H**) Lung metastasis model. The lung sections were subjected to H&E staining, and the metastatic nodules are indicated with arrows. The number of metastatic nodules in the lung specimens was analysed. **p < 0.01.

To further identify the effects of RASSF6 on CRC *in vivo*, we established BALB/c nude mouse xenograft models using the stable cell lines RASSF6 and Vector. Results showed that both the weight and volume of tumours were significantly decreased in the RASSF6 group compared with the Vector group (Figure 5A and 5B). Moreover, we performed immunohistochemistry to examine the expression patterns of RASSF6 and Ki67, a nuclear cell proliferation marker, in the collected tumours. As shown in Figure 5C, tumours from the RASSF6 group displayed a weaker Ki67 staining intensity compared with that of the control groups, and the expression level of RASSF6 negatively correlated with Ki67 expression (*p* < 0.05, Figure 5D).

Next, we established hepatic and lung metastasis models to evaluate the effect of RASSF6 on CRC cell metastasis. For the hepatic metastasis model, cells were implanted into the spleens of nude mice. After 10 weeks of injection, livers were collected from the mice, and the number and size of metastatic tumour nodules were examined by microscopy (Figure 5E). The results revealed that the number of metastasis nodules from the RASSF6 group was significantly less than that of the Vector group (*p* < 0.05, Figure 5F). For the lung metastasis model, cells were injected into the tail veins of nude mice. Ten weeks later, the mice were sacrificed, and the lungs were collected. As shown in Figure 5G and 5H, RASSF6 overexpression led to a dramatic decrease in the number of metastatic nodules, which was consistent with the *in vitro* results described above. Therefore, we conclude that RASSF6 inhibits tumour proliferation and metastasis in colorectal cancer.

## DISCUSSION

Comprehensive knowledge of the genes that drive malignant tumour formation and progression is critical for cancer diagnostics, therapeutics, clinical trial design, and the selection of rational combination therapies. In this study, we demonstrated that RASSF6 is a tumour suppressor in colorectal cancer and may serve as a promising novel therapeutic target for colorectal cancer.

Studies of several tumours have shown that RASSF6 expression is downregulated, and low RASSF6 expression corresponds to a poor prognosis. Chen et al. analyzed the relationship between RASSF6 expression and the clinicopathological features of in 52 CRC patients, and uncovered the correlation of RASSF6 expression with disease stage [19]. However, no study has yet to highlight the importance of RASSF6 in relation to the prognosis of CRC patients.

In our study, our results in function assays are consistent with that reported by Chen et al. which further confirmed that RASSF6 indeed inhibit tumorigenesis in CRC. More importantly, the main innovation points of our study are reflected in following aspects. First, we investigated the underlying molecular mechanisms through which RASSF6 inhibited tumorigenesis in CRC, which enriched and deepened the study reported by Chen et al. Secondly, except for negative correlation between RASSF6 expression and advanced TNM stage, we also found that low RASSF6 expression was significantly associated with tumour size (*p* = 0.042), lymph node status (*p* < 0.001), and the presence of distant metastasis (*p* < 0.001, Table 1), which provided more powerful evidence that RASSF6 suppresses CRC proliferation and metastasis. Thirdly, our results revealed RASSF6 acted as an independent prognostic factor for PFS and OS in CRC, which gain further insight into the prognostic value of RASSF6 expression in CRC. Addtionally, we first established both hepatic and lung metastasis models to demonstrate that RASSF6 inhibits metastasis of CRC cell lines *in vivo* to further confirm the role of RASSF6 in CRC (Figure 5E–5H), which has not been reported before. To the best of our knowledge, our study first emphasized the role of RASSF6 in predicting the prognosis of CRC patients, discovered that RASSF6 suppresses EMT process, and inhibits tumorigenesis of CRC at least in part, through suppressing Wnt signaling pathway.

Epithelial–mesenchymal transition (EMT) is a phenotypic alteration that converts adherent epithelial cells into individual migratory cells that can invade the extracellular matrix, and this process is closely associated with the metastasis and progression of tumours [25, 26]. As RASSF6 inhibited the motility of CRC cell lines *in vitro* and *in vivo*, we investigated whether RASSF6 could exert its function through the regulation of the EMT process. Notably, RASSF6 efficiently repressed both the mRNA and protein expression of the mesenchymal marker Snail and enhanced the expression level of epithelial markers ZO-1 and E-cadherin (Figure 3C–3F); these results are consistent with those of the transwell assays. Thus, we found that RASSF6 inhibits the migration and invasion of CRC cells, which is at least partially due to its ability to repress EMT.

However, the underlying mechanisms accounting for the role of RASSF6 in CRC remain undefined. Because of the crucial role of the Wnt signalling pathway in CRC, we first investigated whether RASSF6 participates in the regulation of Wnt signalling in CRC cells. Consistent with our expectations, the expression levels of c-Myc, c-Jun, and TCF1, all of which are classic Wnt signalling target genes, were significantly decreased in the RASSF6-overexpressing DLD1 stable cells (Figure 3B). Moreover, to further validate whether RASSF6 indeed inhibits CRC tumorigenesis by modulating the Wnt pathway, we also performed rescue assays by treating cells with the Wnt activator LiCl. The exogenous introduction of LiCl endowed CRC cells with an enhanced capability for proliferation and migration (Figure 4A–4D), dramatically reversing the inhibitory effect of RASSF6 on the expression of c-Myc, c-Jun, and TCF1 and thus reversing the EMT process while simultaneously decreasing the expression of ZO-1 and E-cadherin as well as increasing the expression of Snail (Figure 4E and 4F). Collectively, we conclude that RASSF6 suppresses the function of CRC cells at least partially through Wnt signalling.

Based on our results, RASSF6 appears to act as a tumour suppressor in CRC development, and Wnt is a potential mediator for RASSF6-regulated EMT progression. Nevertheless, questions remain unanswered. Dysregulated Wnt signalling is widely implicated in various malignancies [27, 28], especially in colorectal cancer [29], and this leads to cell changes that influence a vast array of biological processes, including embryonic development, cell proliferation [30], cell migration [31], stem cell maintenance [32], and tumourigenesis [33]. Given that RASSF6 inhibiting Wnt signalling in CRC, we suspect that RASSF6 may also involve in other biological processes in CRC, such as stem cell maintenance, angiogenesis, and anti-tumour drug resistance, all of which require further investigation in future studies. Additionally, the canonical target genes mediate the effects of Wnt signalling in normal and diseased cells, and RASSF6 overexpression led to a distinct reduction in the Wnt signalling target genes c-Myc, c-Jun, and TCF1, along with a decrease in cell growth and motility; however, the specific target genes that mediate the ability of RASSF6 to act as a tumour suppressor in CRC have not been elucidated. Furthermore, considering that several signalling pathways participate in the tumour suppressor role of RASSF6 in other types of tumours, there is a strong possibility that Wnt is not a unique mechanism involved in the RASSF6 regulation of CRC. For example, in melanoma, RASSF6 suppresses MAPK activation [17]; whether RASSF6 also inhibits MAPK signalling in CRC, and whether Wnt is the primary mechanism that mediates the regulation of RASSF6 in CRC remains to be determined.

In summary, our findings demonstrate a potential inhibitory role for RASSF6 in CRC tumorigenesis. Our data suggest that RASSF6 may emerge as a new prognostic marker and an effective therapeutic target in colorectal cancer.

## MATERIALS AND METHODS

### Cell line culture

The human colorectal cancer epithelial cell lines HCT116, DLD1, SW620, SW480, Caco2, HT29, and HCT15 were preserved in our Institute, and the human colorectal epithelial cell line FHC was purchased from ATCC. FHC cells were cultured in DMEM:F12 medium (containing 0.005 mg/ml transferrin, 10 ng/ml cholera toxin, 0.005 mg/ml insulin, and 100 ng/ml hydrocortisone). HCT116 was cultured in DMEM with 10% foetal bovine serum (FBS). DLD1 was maintained in RPMI1640 medium with 10% FBS. SW620 cells were cultured in Leibovitz's L-15 medium supplemented with 10% FBS. All cell lines were cultured in a humidified atmosphere with 5% CO_2_ at 37°C.

### RNA extraction and quantitative reverse transcription PCR

Total RNA was extracted from colorectal cells using Trizol (Invitrogen) reagent according to the manufacturer's instructions. A total of 2 μg of RNA was used to synthesize cDNA using M-MLV reverse transcriptase (Promega), and cDNA products were amplified using a SYBR Green PCR Kit (Invitrogen). The primer sequences for q-PCR are summarized in Supplementary Table 1. The relative levels of gene expression are represented as ΔCt = Ct_gene_−Ct_reference_, and the fold-change of gene expression was calculated by the 2^−ΔΔCt^ method. Experiments were repeated in triplicate.

### Western blotting analysis

The total protein of the cells was extracted with cell lysis buffer supplemented with protease inhibitor, and proteins were loaded and separated using 8%–10% sodium dodecyl sulphate-polyacrylamide gel electrophoresis, after which the protein was transferred to PVDF membranes (Roche). Membranes were blocked with 5% non-fat milk and were sequentially incubated with primary and secondary antibodies (Promega). The primary antibodies used were as follows: RASSF6 (Proteintech, 11921-1-AP, 1:1000), TCF1 (Cell Signalling Technology, #2203; 1:1000), c-Myc (Cell Signalling Technology, #5605; 1:1000), c-Jun (Cell Signalling Technology, #9165; 1:1000), β-actin (Santa Cruz, sc-47778, 1:200), E-cadherin (Cell Signalling Technology, #3195; 1:1000), ZO-1 (Cell Signalling Technology, #8193; 1:1000), Snail (Cell Signalling Technology, #3879; 1:1000), and GAPDH (Santa Cruz, sc-365062,1:10000). Finally, the blots were visualized using an electrochemiluminescence system (KeyGEN BioTEC). The western blot bands were quantified by densitometry and normalized to either GAPDH or β-actin which served as internal protein loading control. Results were expressed as fold changes vs. controls.

### Immunohistochemical staining

Patient CRC tissues (*n* = 127) were collected from The Third Affiliated Hospital of Southern Medical University. Paraffin sections were cut to a thickness of 4 μm. All sections were deparaffinized in xylene, rehydrated in successive washes of ethanol, and blocked in methanol with 0.3% H_2_O_2_. The tissue antigens were then retrieved in a microwave oven at medium power for 25 minutes in citrate buffer (pH 6.0). Tissue sections were treated with primary antibodies, which were diluted with background-reducing components (Dako), and then incubated overnight at 4°C in a moist chamber. The antibodies used were as follows: RASSF6 (1:100, Proteintech) and Ki67 (Santa Cruz, sc-15402, 1:200). The slides were sequentially incubated with a secondary antibody for 30 min using the Envision Detection Kit (Dako) at room temperature and were stained with diaminobenzidine tetrahydrochloride (DAB). Finally, the sections were counterstained with haematoxylin, dehydrated, and mounted. A negative control was included by replacing the primary antibody with PBS. Three independent observers who were not informed of the clinical data of the patients evaluated the staining intensity. Their conclusions were in complete agreement in 85% of the cases, which suggested that the scoring method was highly reproducible. If two or all of them agreed with the results they scored, the value was selected. If the results were completely different, then all of them would work collaboratively to confirm the score. The expression of RASSF6 was assessed using H-scores. The samples were grouped into four categories based on the intensity of the staining: none (0), weak (1), medium (2), and strong (3). The H-score was defined by multiplying the intensity score by the percentage of the stained area. A receiver operating characteristic (ROC) curve analysis was applied to determine a cutoff value for RASSF6 low expression and high expression. The sensitivity and specificity for the outcome under study was plotted, thus generating a ROC curve. The score that was closest to the point with both the maximum sensitivity and specificity was selected as the cut-off value.

### Patients and tissue specimens

The specimens used for IHC were collected from 127 patients who were histologically and clinically diagnosed with colorectal cancer by The Third Affiliated Hospital of Southern Medical University between 2004 and 2010. The specimens used conformed to the criteria that they contain matched tumour tissue (percentage of tumour cells 70%) and corresponding normal mucosal tissue (> 5 cm laterally from the edge of the tumour region). The use of clinical specimens for research was approved by the Institutional Research Ethics Committee, and the patients were informed prior to the use of clinical material. Patients who had a single primary lesion and no neoadjuvant chemotherapy were included, and the patients who did not have follow-up information were excluded from this study.

### Cell transfection

The plasmid expressing RASSF6 (pENTER-RASSF6) and empty control vector (pENTER) were purchased from Vigene Bioscience (Shandong, China). We seeded 4 × 10^5^ cells per well in a six-well plate, and after 24 h, we transfected the cells with RASSF6-expressing plasmid or the corresponding empty vector plasmid using Lipopectamine 2000 (Lipo2000) transfection reagent (Invitrogen) according to the manufacturer's instructions. The transfected cells were incubated for 6 h and were refreshed with complete medium containing 10% FBS. The total RNA or protein was collected 48 h after transfection.

### Stable cell line generation

DLD1 cells were transfected with pENTER-RASSF6 or empty plasmids. Cells were harvested at 48 h after transfection, and puromycin was added for 10 days to select stable overexpression cells; the medium was refreshed every 2 or 3 days. For persistent suppression of RASSF6 expression, HCT116 cell lines stably expressing the short hairpin RNA (shRNA) targeting RASSF6 were established using the pSUPER RNAi system according to the manufacturer's instructions. RASSF6 shRNA constructs were synthesized by Invitrogen. The sequences of the shRNAs were as follows: sh1, 5′- GAACAAAGACGACTAAAGA -3′ and sh2, 5′- GGAATTTGACGATCTCTAT -3′. Q-PCR and western blotting was used to evaluate RASSF6 expression.

### Cell viability assays

Cell proliferation was determined by MTT assays and colony formation assays. For MTT assays, the cells were seeded in 96-well plates at 1 × 10^3^ cells per well, and the absorbance of each sample was measured at 490 nm. For colony formation assays, 400 cells were plated into six-well plates and supplemented with complete medium containing 10% FBS. After incubating at 37°C for 10–14 days, the cells were washed with phosphate-buffered saline (PBS), and visible colonies were fixed and stained with 0.5% crystal violet and methanol. The average of 3 repeated experiments was calculated.

### Cell migration and invasion assay

Cell migration and invasion were evaluated using transwell assays. Transwell assays were performed using chambers without Matrigel (8-μm pore; BD Falcon) to test migration or with Matrigel (8-μm pore; BD Falcon) to test invasion. We seeded 1 × 10^5^ cells in serum-free medium into the cell culture inserts, and then complete medium containing 10% FBS was added to the bottom chamber. After 20 h of incubation, the cells attached to the lower surface of the insert filter were fixed, stained with crystal violet, and counted using a microscope.

### Luciferase reporter assay

Cells were seeded into 24-well plates and transfected with 200 ng of TOP-flash/FOP-flash plasmids (Addgene), 20 μg of pRL-TK vector (Promega), and 300 ng pENTER-RASSF6 or pENTER using Lipo2000 transfection reagent. To examine Wnt activity, cells were treated with LiCl (Sigma) and were harvested 48 h after transfection. The luciferase activity was measured using the Dual Luciferase Reporter assay kit (Promega). Experiments were performed three times independently.

### Animal experiments

Male BABL/c nude mice (15–18 g, 4–5 weeks old) were purchased from Slaccas Experimental Animal Center (Shanghai, China). All animal studies were conducted in accordance with the NIH animal use guidelines. For *in vivo* proliferation assays, mice were randomly allocated into two groups (*n* = 7). DLD1 cells stably overexpressing RASSF6 or control stable cells were suspended in PBS, and 1 × 10^6^ cells in 100 μL of PBS were subcutaneously injected into the nude mice. The tumour size was measured every 4 days, and the tumour volume was calculated. After 28 days, mice were euthanized, and the primary tumours were collected and weighed. The tumour volume was calculated using the following formula: V = (width^2^ × length)/2.

For *in vivo* metastasis assays, we established both hepatic and lung metastasis models. For the hepatic metastasis model, mice were randomly allocated into two groups (*n* = 7), anesthetized with isoflurane, and subjected to laparotomy. We injected 1 × 10^6^ cells in 100 μL PBS into the distal tip of the spleen using an insulin syringe. Ten weeks after injection, the mice were euthanized, and the spleens and livers were collected for pathologic analysis. For the lung metastasis model, 1 × 10^6^ cells resuspended in 100 μL of PBS were injected into the tail veins of the mice. Ten weeks after injection, the mice were sacrificed, and the lungs were harvested for pathologic examination. Transverse sections (5 mm) of the liver or lung were prepared, and the sections were stained with haematoxylin and eosin (H&E). Metastatic nodules were counted manually using a microscope.

### Statistical analysis

In our study, all statistical analyses were conducted using the SPSS 16.0 statistical software package. Two-tailed *X*^2^ tests were used to evaluate associations between RASSF6 expression and clinicopathological parameters. Univariate and multivariate analyses were conducted using the Cox proportional hazard regression model to analyse the associations between clinicopathological variables and patient mortality. Survival curves were plotted by the Kaplan-Meier method and were compared using the log-rank test. A *p* value below 0.05 was considered statistically significant.

## SUPPLEMENTARY MATERIALS FIGURES AND TABLES



## Abbreviations

CRC: colorectal cancer
RASSF6: Ras association domain family 6
EMT: epithelial-mesenchymal transition
IHC: immunohistochemistry
shRNA: short hairpin RNA, q-PCR: quantitative-polymerase chain reaction
