# Supplementary material for: RASSF6 downregulation promotes the epithelial-mesenchymal transition and predicts poor prognosis in colorectal cancer
Source: Oncotarget. 2017 Jul 12;8(33):55162–75. doi: 10.18632/oncotarget.19181 (PMC5589649; doi:10.18632/oncotarget.19181)
Supplement: Supplementary file 1 [file oncotarget-08-55162-s001.pdf]

RASSF6 downregulation promotes the epithelial-mesenchymal transition and predicts poor prognosis in colorectal cancer

SUPPLEMENTARY MATERIALS

Supplementary Table 1: Primer sequences for q- PCR

Gene	Sense primer	Antisense primer
RASSF6	ACAGGACCCAGATTCCTATGTC	GCTGCTTCACTCATGGTTCTAT
GAPDH	CCCACATGGCCTCCAAGGAGTA	GTGTACATGGCAACTGTGAGGAGG
ZO-1	CAACATACAGTGACGCTTCACA	CACTATTGACGTTTCCCCACTC
E-Cadherin	CGAGAGCTACACGTTCACGG	GGGTGTCGAGGGAAAAATAGG
Snail	ACTGCAACAAGGAATACCTCAG	GCACTGGTACTTCTTGACATCTG

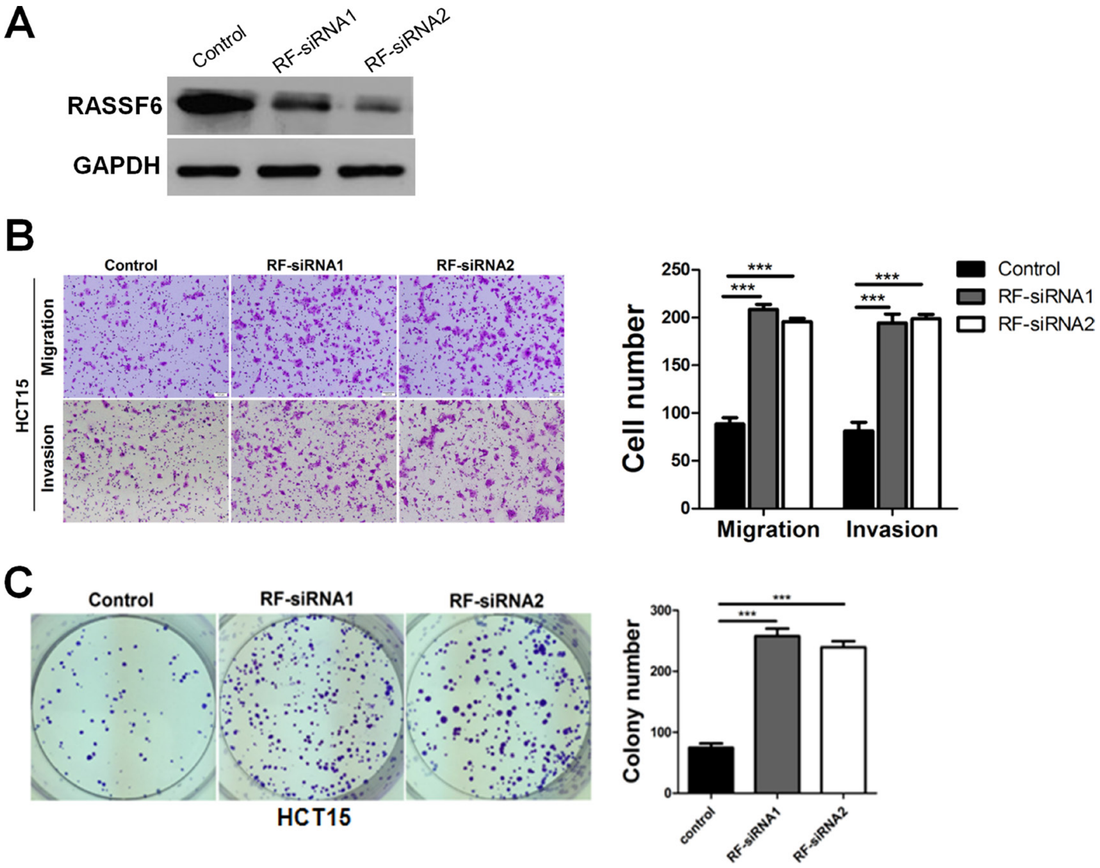

Supplementary Figure 1: Silence of RASSF6 promotes cell growth and motility in HCT15 cells. (**A**) The efficient suppression of RASSF6 was confirmed by western blotting in HCT15 cells. (**B**) Reprensentative images show the migration and invasion abilities of HCT15 cells with RASSF6 transiently knocked down. The number of cells was quantified. ***$p < 0.001$, Student's *t*-test. (**C**) Colony formation assays were performed, and representative images show the proliferation ability of HCT15 cells with RASSF6 transiently knocked down. The number of colonies was quantified. These above experiments were repeated at least three times. Error bars, mean ± SD, ***$P < 0.001$ using Student's *t*-test.
